# Early-Life Host–Microbiome Interphase: The Key Frontier for Immune Development

**DOI:** 10.3389/fped.2017.00111

**Published:** 2017-05-24

**Authors:** Nelly Amenyogbe, Tobias R. Kollmann, Rym Ben-Othman

**Affiliations:** ^1^Department of Experimental Medicine, University of British Columbia, Vancouver, BC, Canada; ^2^Department of Pediatrics, University of British Columbia, Vancouver, BC, Canada

**Keywords:** microbiome, immunity and infections, ontogeny, immune diseases, probiotics

## Abstract

Human existence can be viewed as an “*animal in a microbial world*.” A healthy interaction of the human host with the microbes in and around us heavily relies on a well-functioning immune system. As development of both the microbiota and the host immune system undergo rapid changes in early life, it is not surprising that even minor alterations during this co-development can have profound consequences. Scrutiny of existing data regarding pre-, peri-, as well as early postnatal modulators of newborn microbiota indeed suggest strong associations with several immune-mediated diseases with onset far beyond the newborn period. We here summarize these data and extract overarching themes. This same effort in turn sets the stage to guide effective countermeasures, such as probiotic administration. The objective of our review is to highlight the interaction of host immune ontogeny with the developing microbiome in early life as a critical window of susceptibility for lifelong disease, as well as to identify the enormous potential to protect and promote lifelong health by specifically targeting this window of opportunity.

## Introduction

A key function of the immune system is to interact with and respond to the environment (1). Microbes are a major part of this environment. In fact, all animals harbor diverse, host-specific microbial communities, each discretely assembled in organ-specific microhabitats across nearly all parts of the body (2). Not surprisingly then, the host–microbiome interphase was found to be key for optimal immune function in adults (3, 4). However, emerging data strongly indicate that the most formative period for this interaction occurs very early in life (5–8), possibly starting even before birth (9, 10). One of the first detailed longitudinal surveys of the intestinal microbiota in its first year of postnatal development found a rapidly changing succession of bacterial taxa beginning with aerobes such as *Streptococcus* and *Staphylococcus* in the first week of life that soon are replaced by obligate anaerobes like *Prevotella* and *Veillonella*, which then continue to feature prominently into adult life (11). A later seminal work compared the genetic potential, or microbiome, of babies compared to adults across geographically distinct populations and found that with dramatic shifts in colonization early in life also came functional shifts, while infant bacteria contain folate synthesis genes, those in adults contain more genes for folate metabolism and cobalamin, vitamin B7, and B1 synthesis (12). Since then, the development of microbiota throughout infancy has been the topic of numerous reviews (13–16).

Given the rapid changes of the microbiome in early life (16), even minor perturbances during this highly dynamic phase could have negative long-lasting consequences (17). For example, in atopy, an at least partly immune-mediated disease, alterations of the microbiota in early life appear to be the culprit (18). Specifically, atopic infants harbor fewer Bifidobacteria, Lactococci, and Enterococci as early as 1 week of age as compared to non-atopic controls (19); and infants diagnosed with atopy at 5 years of age had been less likely colonized at 1 week of age with *Bacteroides adolescentis* and Lactobacilli Group I as compared to non-atopic infants (20). Furthermore, colonization at 1 month of age with *E. coli* and *C. difficile* is associated with increased risk of eczema in first 2 years of life, and for *C. difficile* specifically with recurrent wheeze, allergic sensitization, and atopic dermatitis at 2 years (21). How such differences in the microbiota lead to clinically symptomatic atopic disease is not understood, but given the underlying immune pathogenesis of atopy, the mechanisms likely involve altered immune ontogeny (22). One recent study identified newborns with a distinct microbiota enriched in fungal species *Rhodotorula* and *Candida* together with decreased relative abundance of *Bifidobacterium, Lactobacillus*, and *Akkermansia* who suffered an increased risk for atopy at 2 years of age and physician-diagnosed asthma at 4 years of age. Moreover, sterile fecal waters from such at-risk infants induced a higher proportion of IL-4-secreting compared to IFNγ-secreting adult CD4+ cells, linking fecal metabolites to possible immune cell alterations that could play a role in increased asthma risk for these children (23). Other studies have linked particular early-life microbiota to variation in immune ontogeny later in infancy. For example, newborns colonized with *Bacteroides fragilis* express lower levels of TLR4 and TLR2 mRNA in their peripheral blood leukocytes at 1 year of age and produce lower levels of inflammatory cytokines (24). However, in another study, infants with a greater abundance of *Bacteroides dorei* in the microbiota during infancy displayed a higher incidence of inflammatory diseases (25). As these contrasting results involve distinct human populations as well as different strains of *Bacteroides*, they caution against generalizing properties of different, but related microbial species across populations.

Assigning molecular cause–effect relationships to alterations of the microbiome with impact on the developmental trajectory of the immune system is difficult given the complexity of the systems involved and the rapidity with which each changes (5, 26). However, *B*. *fragilis* is an exception as it provides one of the best studied examples of a human commensal driving immune ontogeny. Specifically, polysaccharide A (PSA), a sphingolipid specific to *B. fragilis*, was among the first bacterial products shown to induce maturation of CD4+ T cells in both the mucosa and spleens of germ-free mice (27). *B. fragilis* PSA in particular was shown to play a critical role in neonatal immune development, where colonization with PSA-expressing *B. fragilis* was necessary for regulatory T cell (Treg) development and invariant NKT cell inhibition in the intestine—the absence of which led to exacerbated inflammation in adulthood (28). Importantly, colonizing adult mice with *B. fragilis* failed to correct this defect (28), indicating critical early-life window of susceptibility for the microbiota to educate the immune system (5). This is again reflected on the clinical level, where differences in microbiota at 3 months of age better predict atopic outcome at 1 year than the microbiota collected at 1 year (29), and microbiota at 3 months predict milk allergy resolution at 8 years of age better than microbiota collected at 6–12 months (30). Further evidence for the existence of a critical early-life window was found when studying the effects of early-life microbial exposure on NK cell phenotypes, where conventionalization of germ-free mice at either 1 week or 3 weeks of life resulted in higher splenic IFNγ-expressing CD4 cells, and higher frequencies of NK and NKT cells compared to conventionally housed mice (31). Immune-regulatory genes were also underexpressed in ileal tissues of the same mice after conventionalization at 1 or 3 weeks of age (32). Clostridial species (specifically, *Clostridium* clusters IV and XIVa) have also been shown to induce Treg accumulation in mouse colons if present during specific early-life periods. Colonizing mice with these bacteria at two weeks of age protects them from colitis in adulthood and lowers their systemic IgE levels (33). On the other hand, exposure to segmented filamentous bacteria (SFB) in early life of mice is uniquely able to induce large numbers of Th17 cells (34) using a mechanism dependent on their adherence to the intestinal epithelium (35). Through induction of Th17 cells, SFB were also shown to exacerbate autoimmune arthritis in colonized germ-free mice (36). While the role of SFB, or similar bacteria in the neonatal period has yet to be defined, one survey of SFB abundance across species and ages found SFB to colonize humans by 2 years of age, but could no longer be found after the third year of life suggesting a possible early-life restricted colonization for these bacteria in humans (37).

While much of the necessary detailed knowledge is still amiss, current data clearly support the notion that perturbations of microbiota in the early-life imprint the host immune phenotype for a long time (maybe lifetime) and can manifest as immune-mediated disease later in life. We here extract overarching themes of how pre-, peri-, as well as early postnatal environmental modulators of newborn microbiota associated with changes in immune ontogeny that predispose to disease; given the little data there are on this topic, we focus on those disease states for which existing data suggest this to be a plausible if not reasonable connection. In doing so, we also begin to delineate the windows of opportunity, knowledge of which should help guide to target research efforts into mechanisms and interventions. The goal of this review then is to highlight the potential harm as well as benefit of early-life alteration of the host immune–microbiome interaction and its long-lasting impact on homeostasis and health.

## *In Utero* Colonization Influences Immune Development after Birth

The dogma of a sterile intrauterine environment as necessary for normal, healthy term pregnancies was recently challenged when bacteria were found in human placental membranes (38, 39), amniotic fluid and umbilical cords (40) as well as meconium (41, 42) of healthy term newborns. Even more surprising was the finding that these fetal tissues contained not a random collection of microbes but an organ-specific microbiome. Specifically, the human placenta harbors a unique microbiome with a taxonomic profile that is most similar to the oral cavity of the mother (41, 43). Previously, maternal oral flora had only been thought to be associated with preterm delivery or stillbirth, not healthy term feti (44–46). However, using *in situ* hybridization, bacterial organisms are detectable in 70% of the placental membranes that harbor no sign of inflammation (chorioamnionitis) (39). And in a cross sectional study designed to randomly sample the placental basal plates at delivery revealed *via* histological analysis that of a total of 195 human pregnancies, Gram-positive as well as Gram-negative bacteria of diverse morphologies were detectable in 27% (47). Transmission of bacterial DNA from the oral cavity of the mother to the fetus was directly proven when genetically labeled bacteria orally inoculated into pregnant mice could be detected by PCR in the meconium of the pups delivered by C-section (42). While transmission of maternal microbial products (not live microbes) during pregnancy across the murine placenta to the fetus can be enhanced by the presence of maternal antibodies (10), the mechanism that promotes transfer of live maternal oral flora across the placenta to the fetus has not yet been elucidated.

Given not all the human placentas of term pregnancies examined contained bacteria (39, 47), and the fact that germ-free mice deliver their litter at term (48), it is likely that a placental and fetal microbiome may not be necessary to carry normal pregnancies to term, but serve another function, such as shaping the development of host immune responses in the offspring (9, 49). For example, germ-free newborn mice born to mothers transiently colonized by *E. coli* during pregnancy are better able to avoid postnatal hyper-inflammatory responses and also more readily curtail systemic invasion with intestinal microbes than offspring born to non-colonized dams (10). Maternal colonization appeared to reprogram intestinal transcriptional profiles in the offspring including increased expression of genes encoding epithelial antibacterial peptides as well as metabolism of microbial molecules; gestational colonization also increased intestinal group 3 innate lymphoid cells as well as F4/80+CD11c+ mononuclear cells (10). The data of this study support the notion that the maternal microbiota and its products transferred to the fetus prepare the newborn for optimal host–microbial mutualism, rather than solely enhancing antibacterial immune responses (10).

The findings summarized above suggest that actively modulating the maternal microbiome *via* probiotics during pregnancy may provide avenues to modulate immunity in her offspring. For example, in a randomized double-blind placebo-controlled trial where 29 women who were to undergo an elective C-section at term received *Lactobacilli* and/or *Bifidobacterium lactis* 14 days prior to delivery, the presence of the specific probiotic administered orally to the mother was detectable in the placenta, the amniotic fluid, as well as the meconium of the offspring (50). Furthermore, administration of the probiotic to the mother was associated with changes in the expression of Toll-like receptors (TLRs) in the placenta and the infant meconium (50). In particular, a reduced TLR7 mRNA expression was detected in intestinal samples of infants whose mothers received *B. lactis*, while the combination of *B. lactis* with Lactobacillus GG was associated with decreased TLR6 mRNA expression in the fetal intestine (50). Moreover, oral supplementation with *Lactobacillus rhamnosus* or *B. lactis* probiotics during pregnancy significantly increased cord blood interferon-gamma (IFNγ) production as compared to the placebo group (51). However, given that the presence of bacterial products in fetal tissues was only recently discovered, the relevance of *in utero* colonization for clinical outcomes in humans has not yet been determined.

## Perinatal Medical Interventions Profoundly Alter the Newborn Microbiome with Lasting Impact on Immune Development and Health Outcomes

Delivery mode (cesarean vs. vaginal delivery) and intrapartum antibiotic use represent two rather common perinatal events that significantly alter a newborn’s microbiota, immune ontogeny, and health outcomes even later life (see Tables 1 and 2).

**Table 1:** **Effect of perinatal perturbances on newborn’s microbiota**.

Perturbance	Sampling age	Microbiota trends
Cesarean delivery	First week of life	Fewer *Bifidobacteriaceae, Enterobacteriaceae, Bacteroides*, and *Lactobacilli*, and greater relative abundance of *Haemophilus, Veillonella, Clostridiaceae*, and *Klebsiella* (66)
	First 3 months of life	Fewer *Bacteroidaceae* and greater abundance of *Clostridiaceae* (with more striking differences for emergency vs. elective C-sections) (70)
	First 12 months of life	Fewer *Bacteroidales* other taxa like *Clostridiales* and more abundance of *Enterobacteriaceae* (68)
Intrapartum antibiotic exposure	Day 3 of life	Reduced *Bacteroides* and *Parabacteroides*, increased abundance of *Enterococcus* and *Clostridium* (70)
	Day 7 of life	Reduced bacterial diversity with lower levels of *Bifidobacteria* and *Bacteroides*, and higher levels of *Enterobacteriaceae* or *Streptococcaceae* (77, 78)
	Day 7 and 30 of life	Reduced proportions of *Bifidobacteria* and increased proportions of *Enterobacteria;* no changes in *Lactobacillus* and *Bacteroides* at any time (80)
Neonatal antibiotic exposure	First weeks of life	Increased abundance of *Enterococcaceae* (70, 77–80)
Formula feeding		Reduced abundance of *Bifidobacteria* and *Lactobacilli* (85)

### Cesarean Delivery (CD)

Cesarean deliveries have increased globally from 6.7% in 1990 to 19.1% in 2014, with rates above 30% in several countries such as the United States, Brazil, and China (52). While CD can certainly be lifesaving for indications such as placenta previa and uterine rupture, the growing use of CD has been under increasing scrutiny as data suggest that increased use of elective primary cesareans for low-risk pregnancies can be associated with increased morbidity and mortality to mother and child compared to spontaneous vaginal delivery (53). Further, and more relevant to this review, CD has been associated with range of immune-mediated diseases in the offspring, such as an increased risk for type 1 diabetes (54, 55), celiac disease (55), childhood and adult obesity (56–58), asthma (59, 60), and allergic disease (61, 62). CD may also be associated with susceptibility to infections, as CD born infants are more likely to be hospitalized for bronchitis throughout the first 2 years of life (63). In all this, the microbiota has often been implicated as a driver of these various immune-mediated diseases.

The vagina provides vaginally delivered (VD) newborns with their first *ex utero* microbial inoculum. The skin and oral microbiota of VD newborns moments after birth, and rectum 24 h after birth, closely resembles the mother’s vaginal microbiota (64). In contrast, CD infants’ microbiota most closely resembles skin microbes and is no more like their mother’s than to another women’s skin microbiota. For example, in Swedish infants and their mothers, 72% of operational taxonomic units (a DNA sequence-based classification of bacteria) detected in stools of VD infants at 1 week of age could also be found in the mother’s stool; this was reduced to only 40% for CD infants (65). In a recent meta-analysis, microbiota of CD newborns was found to be less diverse within the first week of life, harbored fewer *Bifidobacteriaceae, Enterobacteriaceae, Bacteroides*, and *Lactobacilli*, and greater relative abundance of *Haemophilus, Veillonella, Clostridiaceae*, and *Klebsiella* than VD infants (66). Furthermore, increased abundance of *Clostridiaceae* was detectable up to 2 months, and both lower diversity and relative abundances of *Bifidobacteria* and *Bacteroides* were detectable up to 3 months of age. However, the microbiota of CD and VD infants became increasingly less distinguishable over the first 3 months of life, suggesting an equalizing influence of the environment. This has again been noted in more recent studies, where the microbiota of infants differed by mode of delivery at birth for the nares, mouth, and skin but not for meconium, with few differences still seen at 6 weeks of age (67). And another survey of 24 VD and 19 CD newborns showed that while the stool microbiota of both groups converged by 2 years of age, CD infants were less colonized by *Bacteroidales* during the first year of life, while other taxa such as *Clostridiales* and *Enterobacteriaceae* became more abundant (68).

It is interesting to note that microbiota of elective vs. emergency cesarean deliveries can often be not distinguished. Only one small study reported lowest bacterial diversity among three infants delivered by elective CD compared to three infants delivered by emergency CD, which were more similar to VD infants (69). In another study, the skin, nares, mouth, and meconium or stool microbiota of infants were surveyed alongside their mothers at the same four sites in addition to the vagina at birth or 6 weeks of age (67). Here, the differences seen by delivery mode at birth were most apparent for CD infants born without labor, compared to CD or CD after labor onset. However, the sample size of this study also was limited, as only 13 mother–infant dyads were sampled at the 6-week time points for combined labored- and unlabored-CD compared to 40 VD diads. In a more highly powered study comparing 17 elective CD, 23 emergency CD, 40 VD infants born to mothers given intrapartum antibiotic prophylaxis (IAP), and 96 VD infants not exposed to any antibiotics found the opposite, namely, that both elective and emergency CD infants harbored fewer *Bacteroidaceae* and greater *Clostridiaceae* at 3 months of life compared to VD infants irrespective of IAP exposure, but these differences were more striking for emergency CD infants rather than elective. Moreso, these differences persisted up to 1 year of age more in emergency CD infants compared to any other group (70). As such contradictory findings may be due to sample size, larger cohorts are needed to provide more insight into colonizing differences between elective vs. emergency CD infants, together with changes due to antibiotic use alone—especially since the effect of CD on the microbiota overall has been minimal—explaining only 2% of total variance in the first year of life (68) and less than 4% even at birth (67). Large, well-defined cohorts will be necessary to capture these differences.

However, such equalization was not seen for immune responses, where differences between CD and VD infants remain detectable up to 2 years of age. For example, human CD newborns harbor fewer IgA, IgG, and IgM-secreting cells throughout the first year of life (71), as well as lower levels of Th1-supporting chemokines CXCL10 and CXCL11 (72), lower levels of IFNγ and IL-8, and lower CD4+ T-cell responses to tetanus toxoid (73) over the first 2 years of life. Mouse studies further support imprinting of immune differences in the immediate period after CD vs. VD. Mice delivered by CD display distinct microbiota at weaning, but not later in adulthood. On the contrary, immune differences persist from the newborn period into adulthood, where CD mice display a lower tolerogenic mucosal immune profile with fewer Tregs and *IL10* gene expression in their mesenteric lymph nodes as compared to VD mice (74).

In summary, while the epidemiological data regarding a causative links between CD and any of the aforementioned immune-mediated diseases were not drawn from randomized trials and have yet to be confirmed using relevant animal models, CD infants appear to display an increased risk to suffer from several immune-mediated diseases. The evidence that CD born infants display an immune developmental trajectory that differs from VD born infants on the other hand is sound. Equally robust is the finding that microbiota of CD infants differs substantially from that of VD infants, but more so during the first 3 months of life, after which differences become increasingly less apparent. Thus, if the microbiota were to represent the mechanistic link between CD, altered immunity, and with that increased disease susceptibility, then early-life differences must have been imprinted during an early-life “window of susceptibility.” However, such window and its associated mechanistic links have yet to be defined sufficiently well in the human setting to address it clinically.

### Intrapartum Antibiotics

Intrapartum antibiotic prophylaxis is the intravenous administration of penicillin or ampicillin to women during labor, who were found to be vaginally or rectally colonized with group B Streptococcus (GBS). Prior to its routine use in the 1990s, early-onset GBS was a leading cause of newborn morbidity and mortality in the United States (75). Following implementation of IAP, early-onset GBS disease incidence fell from 1.7 per 1,000 live births in the 1990s to 0.37 per 1,000 live births by 2008 (76).

Beyond the clinical success of IAP in the prevention of GBS infection in the newborn, the impact of IAP on the newborn infant microbiome has barely been investigated, despite the obvious implications. Most surveys of microbiota alterations due to IAP have been conducted by on group (77–80). This group recruited a cohort of mothers receiving ampicillin for GBS prophylaxis alongside GBS negative mothers not receiving any antibiotics at or within a month of delivery. Stools from their newborns were collected at postnatal days 7 and 30. A fist set of studies compared microbiota of 10 IAP to 10 controls at postnatal day 7 using sequence-based approaches, finding that IAP infants displayed reduced bacterial diversity, lower levels of *Bifidobacteria* and *Bacteroides*, and higher levels of *Enterobacteriaceae* or *Streptococcaceae* (77, 78). In a follow-up study comparing effects of exclusive breast- to mixed-feeding at both 7 and 30 days, microbiota of 13 IAP and 13 controls were assessed with a sequence-based approach, finding that differences between IAP and controls were more prominent in exclusively breastfed (BF) compared to mixed-fed babies, and moreso at 7 days compared to 30. However, IAP infants had reduced proportions of *Bifidobacteria* and increased proportions of *Enterobacteria* regardless of feeding group. And while *Bifidobacteria* proportions equalized by day 30, exclusively BF IAP infants still had especially high proportions of *Enterobacteria* compared to unexposed infants (80). The highest-powered study compared 35 IAP to 49 control infants at days of life 7 and 30 using quantitative PCR for select bacterial taxa, finding fecal bacterial counts of *Bifidobacterium* alone were reduced at day of life 7 only, while *Lactobacillus* and *Bacteroides* were unaffected at any time (79). Only one other group has described alterations of the microbiota due to IAP, and did so for a cohort of Canadian infants receiving IAP either for GBS prophylaxis or CD, and compared their microbiota at 3 months and 1 year of life, finding that IAP was also associated with lower *Bacteroides* as well as lower levels of *Parabacteroides* but higher *Enterococcus* and *Clostridium* levels at 3 months of life among both vaginally and cesarean delivered infants (70). The major differences to persist to 1 year of life were among emergency CD infants only (as discussed above), whereby VD infants were now indistinguishable by IAP exposure aside from a minor increase in *Clostridiaceae*. There is only one study that assessed effects of IAP on the whole genetic content of the microbiota using whole-genome sequencing (67), with few differences found overall, yet functional pathways in the stool at 6 weeks of age revealing correlations to IAP among delivery mode, feeding, maternal weight, and gestational age.

The impact of IAP on immune ontogeny has to our knowledge not been addressed at all. Furthermore, despite the striking similarity of the changes of the microbiota in infants exposed to IAP and those born by CD, and the many health implications associated with CD, the clinical impact of IAP on health outcomes other than neonatal GBS infection has not been addressed at all. The first study addressing this serious knowledge gap is currently in progress, following 240 mother–infant pairs prospectively, assessing IAP and control infant microbiota at 3 months and 3 years of age (81).

In summary, IAP has undoubtedly prevented many newborn GBS-related deaths. However, given that 10–30% of women in North America are colonized with GBS and receive IAP during labor (82, 83), there has been a surprising lack of effort to address long-term effects of IAP on immune development and the health of their offspring.

## Early Postnatal Events Dramatically Alter the Microbiome with Impact on Long-Term Health Outcomes, but a Causative Role of Immune Changes in This Remains Unexplored

Feeding mode (breast vs. formula) as well as antibiotic exposure during the neonatal period (here defined as up to day of life 28) have clearly been linked to changes in microbiota (Table 1); their causal relationship to immune development and clinical outcome have surprisingly not been well delineated (Table 2).

**Table 2:** **Effect of perinatal perturbances on newborn’s health**.

Perturbance	Health condition and/or disease associated	Age at onset	Reference
Cesarean delivery	Type 1 diabetes, celiac diseases, childhood and adult obesity, asthma, allergic disease, bronchitis	First 2 years of life to adult life	(54–63)
Antibiotics exposure (before 6 months of age)	Increased risk for corticosteroid-treated wheezing, necrotizing enterocolitis, late-onset sepsis, early mortality, obesity, and exacerbation of hypersensitivity to pneumonitis	First year of life—school age	(108–113, 117)
Formula feeding	Increased risk for diarrheal disease, mortality, diabetes, and overweight. Possible association with a higher occurrence of early-onset inflammatory bowel disease, atopic disease, and ankylosing spondylitis when compared to breastfed infants	First year of life up to 8 years of life	(84, 97, 103)

### Feeding Mode

Differences in the microbiota of BF and formula-fed (FF) infants were first reported nearly 100 years ago with compounds in breast milk found to promote the growth of *Bifidobacteria* (this “bifidus factor” is now recognized as human milk oligosaccharides) (84). As a result, BF infants harbor more *Bifidobacteria* and *Lactobacilli* in their colons than FF infants (85). Interestingly, there is little effect of mixed vs. exclusive BF on the microbiome, as the profound shift in microbiota to an adult-like composition occurs not with the addition of solid food, but rather at cessation of BF (65).

Immune protective functions provided by breast milk were first reported in the 1970s (86). While immunoglobulins were among the first immune molecules recognized in breast milk, breastfeeding has further profound anti-inflammatory influences mediated largely by high concentrations of TGF-β and IL-10, and other immunomodulatory influences mediated by molecules such as soluble CD14, defensins, lactoferrin, and lysozymes that survive passage to the intestinal tract and together act to maintain homeostasis in the colonizing gut (87–90). Indeed, one study has found higher concentrations of anti-inflammatory TGFβ and lower concentrations of pro-inflammatory TNFα and IL-2 in sera of BF compared to FF infants throughout the first year of life (91).

Breast milk contains its own microbiome, harboring a wide range of microbes from 100 to 10^5^ CFU per ml depending on the study (92), with *Streptococcus* and *Staphylococcus* being most common, but others such as *Lactobacillus, Bifidobacterium, Enterococcus*, and *Propionibacterium* readily isolated from milk of healthy women (92). Many short-chain fatty acid producing bacteria such as *Veillonella, Propionibacterium*, and *Faecalibacterium* have also been isolated from breast milk (92). Breast milk itself contains lactic acid bacteria, double-stranded RNA from which stimulates intestinal dendritic cells *via* TLR3 to produce IFNβ, which in turn promotes an anti-inflammatory environment and protects mice against colitis (93, 94). However, the key mediator of the immune homeostatic function of breast milk is presumed to relate to its impact on gut microbiota. In mice, for example, the presence of maternal sIgA in early-life molds the composition of the gut microbiota long into in adulthood, with pups born to sIgA-deficient dams harboring more *Pasteurellaceae* and *Lachnospiraceae* than controls (95).

Many of breast milk’s health-promoting properties were recognized starting over 100 years ago when BF infants were found to suffer less diarrheal disease and reduced mortality rates, and reduced risk for diabetes, and overweight compared to FF infants (84). These were recently reviewed in great detail (87). Beyond diarrhea, BF has since been found to protect from other infections, and in BF newborns who did not receive antibiotics prior to weaning, every additional month of breastfeeding is associated with a 5% decrease in number of postweaning antibiotic courses (96). A recent meta-analysis summarized the powerful evidence that BF is associated with decreased risk for infectious diseases and mortality (97). Specifically, BF infants have only 12% of the risk of FF infants to die in the first 6 months of life. Other data further support an immune-mediated mechanism of BF as one of the possibly responsible mechanisms. For example, BF is associated with lower risk for eczema and recurrent wheeze in first year of life (98), with exclusive BF for >4 months associated with reduced risk for asthma up to 8 years of age (99), and in another study breastfeeding for less than 4 months was associated with increased corticosteroid-treated wheezing episodes in the first year of life (100). However, it is important to note that while meta-analyses do detect a protective effect of BF on asthma and allergic rhinitis, these effects are weaker when limited to studies with the lowest risks of confounding (97, 101). And in a cohort of familial ankylosing spondylitis patients and their families, disease prevalence was 25% in children who were breast fed while it was 40% in the FF comparator (102). Finally, while BF is weakly associated with decreases in early-onset inflammatory bowel diseases but with non-significant differences found for ulcerative colitis and Crohn’s disease separately (103), it is protective against Crohn’s disease-related surgery later in life (104).

Despite the many documented clinical benefits of BF, as well as the known profound impact on immune ontogeny and the microbiome, direct cause–effect relationships between BF-induced changes in the microbiome leading to immune-mediated clinical benefit have not yet been provided.

### Antibiotic Exposure in the Neonatal Period

Empiric antibiotic treatment (EAT) is often given to newborns at risk of developing early-onset sepsis (EOS). Clinical diagnosis of EOS is imprecise and based on non-specific signs and symptoms; rapid, sensitive tests to differentiate infected from uninfected newborns are also lacking (105). Therefore, EAT is administered to a very large number of newborns (106, 107). While this empiric approach can readily be justified given the potentially horrific outcome of treatment delay in EOS (105), the impact on the microbiome, immune development, and clinical outcome beyond sepsis has barely been investigated. The little that is known suggests a profound alteration of normal physiology may occur. For example, antibiotic administration in early life is associated with being overweight at age 12 years (108). Contrary to the previous study that only found associations between overweight and antibiotic use throughout the first year (108), in another study antibiotic use in infants less than 6 months was associated with obesity in childhood, but antibiotic use after 6 months of age was not (109). Moreso, antibiotic administration specifically in the neonatal period was associated with an increased risk for corticosteroid-treated wheezing in the first year of life (100) and allergic rhinitis in school age children (110). Longer duration of antibiotic use in premature infants has been associated with increased risk for necrotizing enterocolitis, late-onset sepsis, and death in early life (111, 112). Even the choice of antibiotic regimen has effects, where ampicillin combined with cefotaxime was associated with increased mortality as compared to ampicillin with gentamicin (113).

As early-life antibiotic use has become a topic of increasing interest, mouse studies have begun to reveal possible cause–effect relationship between early-life antibiotic and later life disease: administration of penicillin to pregnant dams right before birth and through weaning increases body mass of the pups in adulthood, and transferring such perturbed microbiota to germ-free mice is sufficient to replicate this phenotype (114). An association of early-life antibiotic use and altered immune ontogeny is suggested by findings in mouse models where mice exposed to antibiotics prenatally and shortly after birth had increased susceptibility to Vaccinia virus infection and altered CD8 T cell responses at 2 weeks of age (115). Antibiotic exposed infant mice also harbored a microbiota rich in *Enterococcus faecalis* (115), consistent with findings above where human newborns born to mothers given IAP had a microbiota enriched in *Enterococcaceae* (70, 77–80). Further, a series of studies exposing mice to vancomycin in drinking water through pregnancy and weaning exacerbated asthma in pups after weaning (116), an effect that was later linked to greater numbers of eosinophils and neutrophils in bronchoalveolar lavage fluid, increased serum IgE, and reduced frequency of colonic regulatory T-cells (117). While intranasally administered streptomycin had little effect on asthma, it exacerbated hypersensitivity pneumonitis and increased IL-17 and IFNγ expression in the lung (117). It is important to note that while these mouse studies are informative, none of these capture the dose, frequency, or route of neonatal antibiotic exposure seen in humans. Furthermore, while these murine studies suggest a possible connection along the microbiome–immune–clinical outcome axis (which is the topic of this review), studies to investigate this in the human setting have to our knowledge not been conducted.

## Conclusion

**Figure 1:**
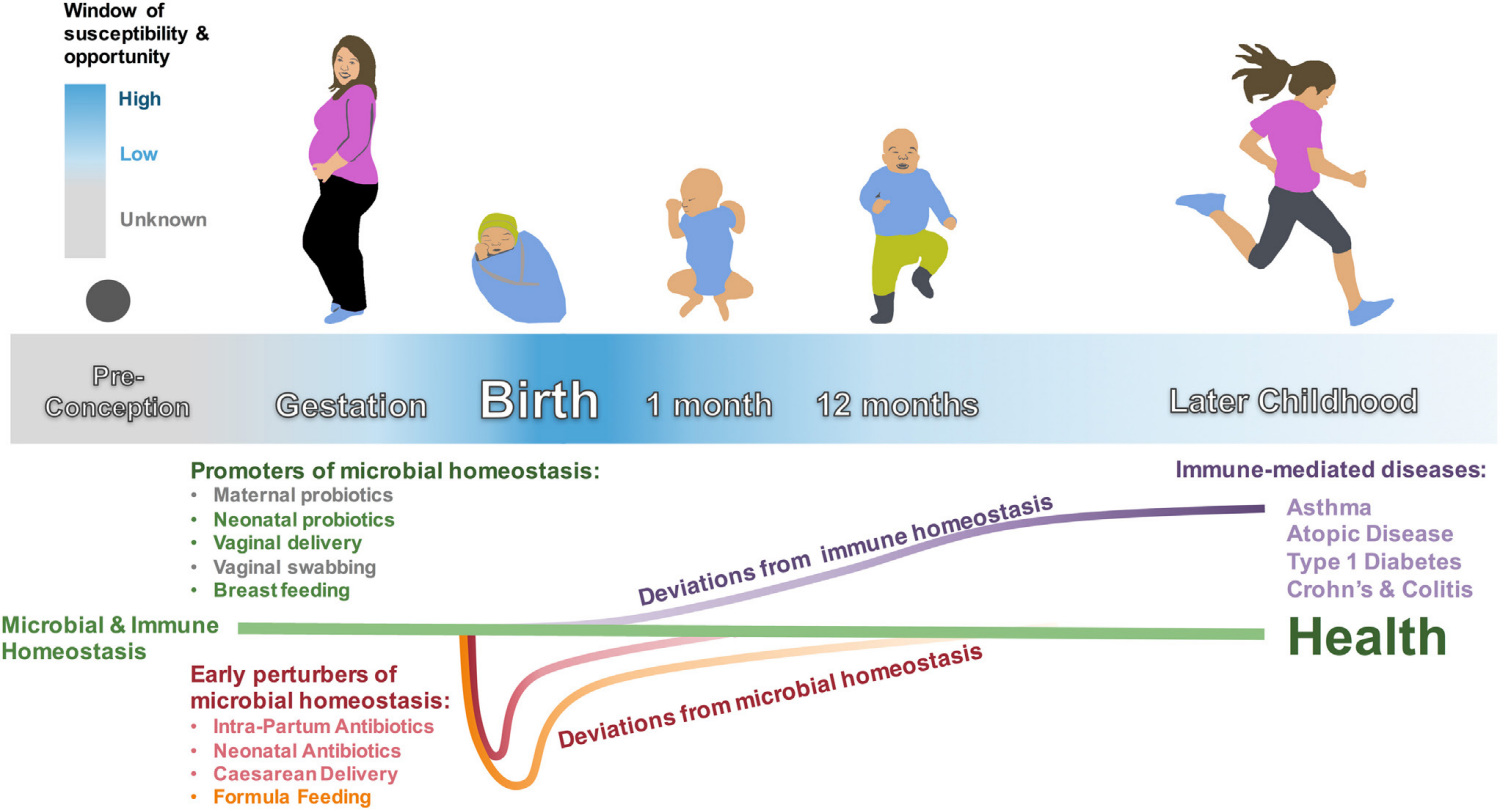
**Microbial and immune homeostasis from preconception to early childhood**. Early-life perturbances (intrapartum antibiotics, neonatal antibiotics, cesarean delivery, and formula feeding) are associated with colonizing differences in the intestinal microbiota that are mostly evident in the first weeks to months of life—with the exception of feeding mode, which is associated with a unique microbiota until cessation of breastfeeding. These perturbers are also associated with immune dysfunction and immune-mediated diseases that manifest later in childhood. The window of susceptibility and opportunity represents the period around birth when promoters of microbial homeostasis may have the largest effect on correcting microbial dysbioses, with an unknown extension into gestation and possibly even preconception. Neonatal probiotics, vaginal delivery, and breastfeeding have strong associations with healthy colonization and decreased risk for immune-mediated disease. Maternal probiotics and vaginal swabbing are possible interventions that need to be further studied. Early-life microbiota and immune-mediated disease in later life need to be studied for cause and effect relationships.

It has been over 100 years that Elie Metchnikoff has popularized the notion of a healthy microbiome as important for a healthy human existence (118, 119). Over the last decade in particular, it has increasingly been recognized that much of this health-promoting interaction is mediated *via* interaction of the microbiome with the human immune system (3, 4, 13, 120–122). Not surprisingly then, perturbations of this evolutionary conserved, beneficial interaction increase the risk for several immune-mediated diseases (17). Emerging now is the concept of an early-life window of increased susceptibility, during which perturbations of this immunity–microbiome interaction cause the most severe and long-lasting damage (5–10). In other words, perturbation of this host–microbiome interphase in early life has to be viewed as a “newborn disease with childhood/adult onset” (Figure 1). With this view in mind, and as reviewed here, it is disturbing to realize that many of these early-life disease-causing perturbations are in fact “man-made,” such as CD, FF, IAP, and EAT. On the other hand, this realization provides us with the opportunity not only to take control and change these choices but also to design well-informed interventions to counteract these perturbations, which are often life-saving and cannot be avoided. In doing so, we can turn the window of susceptibility into a window of opportunity *via*, e.g., timely administration of probiotics (Figure 1) (29, 121–124).

The interaction of the developing microbiome with the host is clearly highly complex, and much of it is currently still unknown (5). But given that the impact of perturbations of the host–microbiome interaction affect clinical outcome far beyond the period, an altered microbiome is detectable suggests the mechanisms involved imprinted themselves into the host in ways beyond the microbiota. In part at least, this relates to the finding of such perturbations often manifesting themselves as immune-mediated diseases; the immune system after all is equipped with long-term memory within both the adaptive as well as innate immune system (125). Innate immune memory already is known to relate to epigenetic alterations (125). However, long-lasting changes in the epigenetic make up of the host in response to alterations of the microbiome extend even beyond the immune system to affect, e.g., metabolism, and connects the theme of this review to the developmental origin of health and disease (126). Specifically, this includes bacterial products that function as substrates for one-carbon metabolism (e.g., vitamins B2, B6, B9, and B12), and substrates for epigenetic modification (e.g., vitamin B7 for biotinylation and vitamin B5 for acetylation), or metabolites that interfere with the host epigenetic machinery (e.g., SCFA-mediated histone deacetylase inhibition) (127). Furthermore, pre- and early postnatal life is thought to be critical window for epigenetic modification specifically because growth and cell division are then at their highest rate. As such, dividing cells require larger amounts of methyl donors to retain cellular methylation patterns that would otherwise be diluted out. Furthermore, bacterial SCFAs such as butyrate and propionate can function as histone deacetylase inhibitors (128), and the *Bacteroides* genus is a major source of propionate in the gut (129). As outlined above, *Bacteroides* colonization is delayed in CD infants and their abundance is reduced in newborns of IAP-treated mothers. While SCFA levels in stools have yet to be investigated in term newborns, propionate levels were found to be reduced in colons of VD piglet colons compared to CD piglets (130). This supports the possibility of a far-reaching impact of the early-life microbiota on our epigenome. On the other hand, such a far-reaching and long-lasting impact also predicts that targeted interventions are likely to have broadly beneficial and long-lasting benefit. For instance, a small study has shown promise that inoculating neonates born by elective cesarean section with vaginal secretions from their mothers leaves them with a microbiota more similar to VD infants compared to infants born by CD that were not inoculated (131). And enteral probiotics administered to premature newborns reduce not only the risk of necrotizing enterocolitis but broadly reduce infection-related mortality (132–138).

From our review of this topic here, several overarching insights can be extracted that help guide future research and intervention efforts:

1. The earlier in life the perturbation, the more profound the impact (both in terms of range as well as duration) (Figure 1) (5–10, 17). This suggests that interventions (e.g., probiotics) would have the most beneficial impact administered as early as possible [e.g., prenatally to the mother (50, 51)].
2. Different perturbations (e.g., cesarean delivery, formula feeding, and intrapartum antibiotic prophylaxis) merge toward a similar final common that often is immune mediated. This suggests that interventions targeting these pathways will likely provide far-reaching, broadly beneficial benefit.

Future research priorities:

1. Impact of prenatal microbiota and viability of organisms found in placenta and amniotic fluid.
2. Understanding effects of cesarean delivery: elective vs. emergency, medical indications, primary vs. repeat, etc.
3. Antibiotic use: reasons for antibiotic administration, comparison to suitable control groups to minimize possible frailty bias. Animal models with comparable exposures to human use.
4. Impact of perinatal events such as chorioamnionitis, neonatal sepsis, and necrotizing enterocolitis on immune and microbiome development.
5. Long-term health impacts of probiotic use in preterm infants.
6. Not discussed in this review is the insight that this interaction of host–microbiome is not restricted to bacteria in the gastrointestinal tract, but also includes fungi, viruses, and other microbes across many other body sites (2).

The complexity of the host microbiome–immunome interaction is astounding, but likely will be deciphered using modern tools of systems biology. The future of this field of study is poised to finally bring about the revolution that Elie Metchnikoff already brilliantly foreshadowed over a century ago (118, 119).

## Author Contributions

NA, TK, and RB-O have reviewed the literature and co-wrote the manuscript. TK and RB-O contributed equally.

## Conflict of Interest Statement

The authors declare that the research was conducted in the absence of any commercial or financial relationships that could be construed as a potential conflict of interest.
